# Precision Medicine in Control of Visceral Leishmaniasis Caused by *L. donovani*

**DOI:** 10.3389/fcimb.2021.707619

**Published:** 2021-11-09

**Authors:** Eduard E. Zijlstra

**Affiliations:** Clinical Sciences, Rotterdam Centre for Tropical Medicine, Rotterdam, Netherlands

**Keywords:** precision medicine and public heath, visceral leishmaniasis, diagnosis, treatment, PKDL, asymptomatic infection, transmission and infection, immune responses

## Abstract

Precision medicine and precision global health in visceral leishmaniasis (VL) have not yet been described and could take into account how all known determinants improve diagnostics and treatment for the individual patient. Precision public health would lead to the right intervention in each VL endemic population for control, based on relevant population-based data, vector exposures, reservoirs, socio-economic factors and other determinants. In anthroponotic VL caused by *L. donovani*, precision may currently be targeted to the regional level in nosogeographic entities that are defined by the interplay of the circulating parasite, the reservoir and the sand fly vector. From this 5 major priorities arise: diagnosis, treatment, PKDL, asymptomatic infection and transmission. These 5 priorities share the immune responses of infection with *L. donovani* as an important final common pathway, for which innovative new genomic and non-genomic tools in various disciplines have become available that provide new insights in clinical management and in control. From this, further precision may be defined for groups (e.g. children, women, pregnancy, HIV-VL co-infection), and eventually targeted to the individual level.

## 1 Introduction

**Figure 1:**
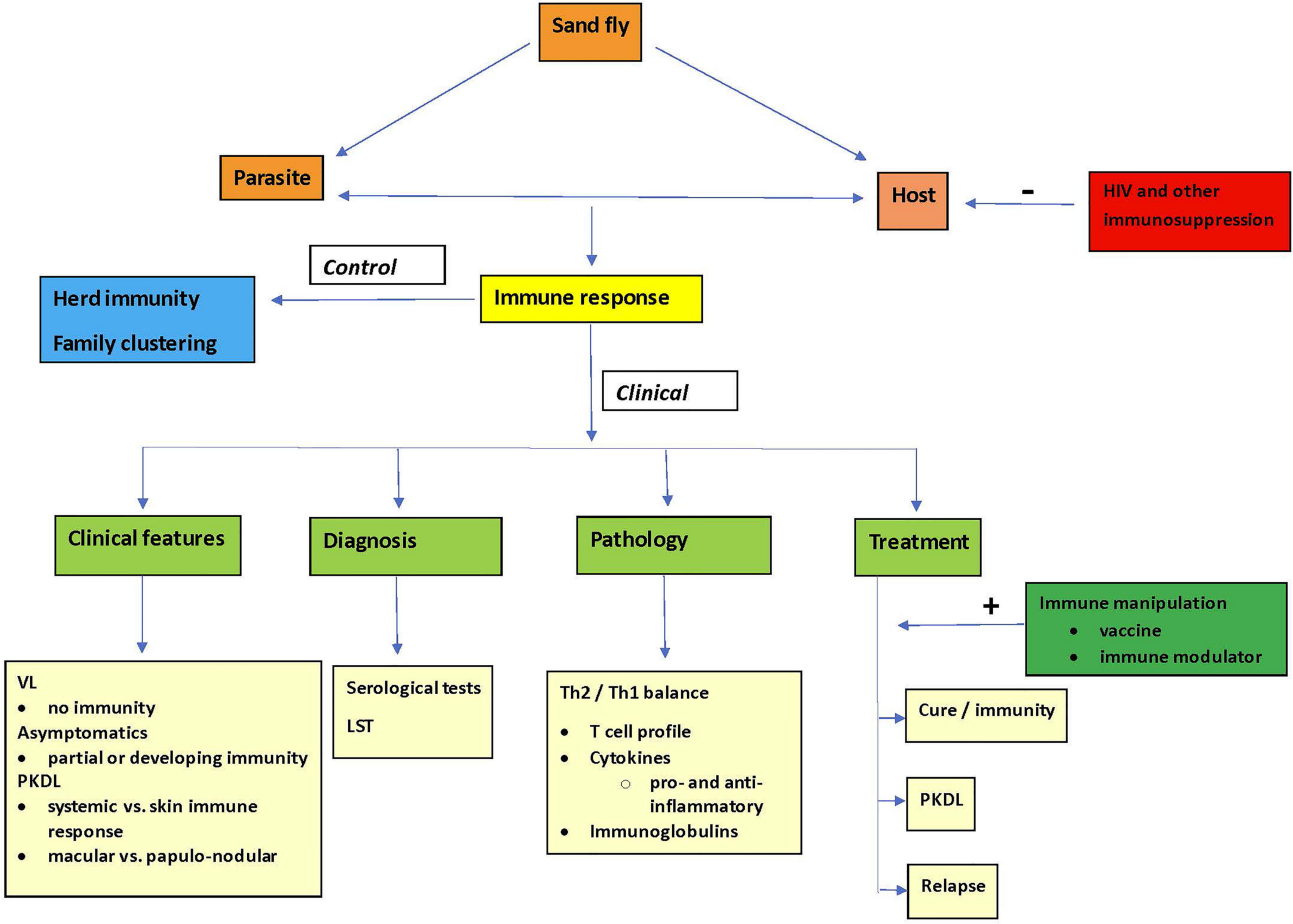
The pivotal role of the immune response in control and clinical aspects of visceral leishmaniasis.

Visceral leishmaniasis (VL or kala-azar) is caused by protozoal *Leishmania (L.) donovani* or *L. infantum* parasites that are transmitted by sand flies of the *Phlebotomus* spp. (Old World) or *Lutzomiya* spp. (New World). There may be anthroponotic and/or zoonotic transmission. The burden of VL is principally in 6 countries (India, Bangladesh, Sudan, South Sudan, Ethiopia, Brazil) (Alvar et al., 2012). While VL is a parasitic disease, virtually all features in clinical presentation, pathophysiology, diagnosis, treatment, outcome as well as epidemiology (herd immunity, family clustering) are determined by the host immune response, that, among other factors, is dependent on the genetics of the host, and in response to the challenge by the causative *Leishmania* parasite and relevant the sand fly vector (**Figure 1**).

Post-kala-azar dermal leishmaniasis (PKDL) is a skin condition characterized by macules, papules, nodules or polymorphic lesions that develop after successful treatment for VL around persisting parasites in the skin, while systemically parasites may no longer be demonstrable in an otherwise healthy individual. It is an intermediate disease state that precedes complete cure from VL. The clinical features are determined by the immune responses. PKDL is thought to play an important role in transmission and is largely restricted to VL caused by *L. donovani* (Zijlstra et al., 2003).

In control efforts, there are important regional differences: with the spectacular reduction of VL cases in the Indian Subcontinent (ISC) due to the successful Kala-azar Elimination Program (KAEP), Africa now has equal numbers of VL cases (Rijal et al., 2019; World Health Organization, 2020). In neighboring Ethiopia HIV VL co-infection is common that may contribute considerably to transmission (Diro et al., 2014). It is likely that the approach to control in each endemic area is guided by regional specific factors; there is no ‘one size, fits all’ in diagnostics, therapeutics or other determinants of control. There are serious gaps in understanding transmission dynamics leading to lack of precision.

### 1.1 Precision Medicine and Precision Public Health

While personalized medicine seeks to utilize treatments and preventive strategies tailored to the individual on the basis of genetic traits only, precision medicine was defined later as the use of diagnostic methods and treatment targeted to the needs of the individual patient(s) on the basis of genetic, lifestyle and environmental determinants.

Human genetics is increasingly contributing to existing research tools, at the level of population, at the level of individual and even to the level of a single-cell e.g. a lymphocyte in relation to a wide array of its immunological properties. This is due the emergence of recombinant DNA and molecular genetic tools including next-generation sequencing of whole genomes and exomes (Neu et al., 2017).

Precision public health (or precision global health) refers to the population perspective and includes applying methods and technologies to measure disease, pathogens, behaviors and susceptibility, as well as developing policies and targeted implementation programs (Singh et al., 2020). Basically, it has been defined as ‘providing the right intervention to the right population at the right time’ (Khoury et al., 2016).

**Figure 2:**
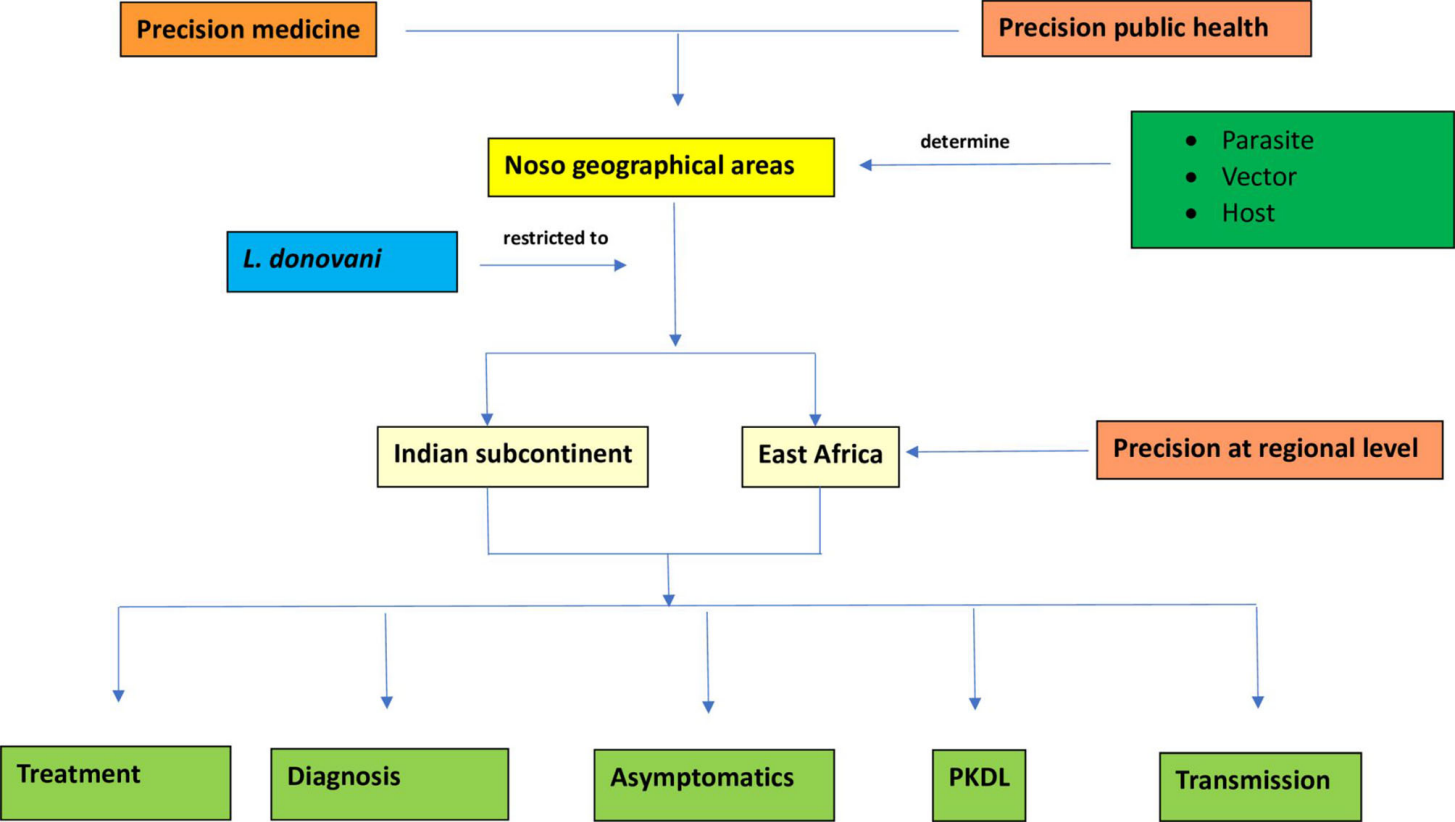
Flowchart describing precision at regional level in visceral leishmaniasis caused by L. donovani through five determinants: treatment, diagnosis, asymptomatics, PKDL and transmission.

In this paper we introduce the concepts of precision medicine and global health to define new insights in VL clinical management and more efficiency in control efforts (**Figure 2**).

## 2 Methods

In absence of publications on precision medicine and global health in VL, relevant literature (general or on VL) was searched for following keywords: genetics, genomics, epigenetics, transcriptomics, nanotechnology, proteomics, metagenomics, pharmaco-genomics, pharmaco-economics, precision medicine and precision global health. The results were first analyzed in factors relating to the host, the parasite and the vector in VL. From this, current and new insights are discussed in the context of existing pitfalls in each of the five main areas relevant for VL clinical management and control: diagnosis, treatment, asymptomatic infection, PKDL and transmission, by discussing new tools (genomic or non-genomic) and re-assessing existing tools. Lastly, a synthesis was attempted to define priorities in precision medicine and global health in VL at the regional level, and where appropriate, at the group or individual level, and suggest the way forward (Flahault et al., 2017; Flahault and Salathe, 2017).

## 3 Results and Discussion

### 3.1. Factors Relating to the Host, Parasite, and Vector

#### 3.1.1 The Host

Genetic studies in VL have indicated that the polymorphic HLA-DR-DQ region within the major histocompatibility complex of immune related genes is the single major determinant of VL. This was found in a study that assessed three independent cohorts in *L. donovani* in India and *L. infantum/chagasi* in Brazil; a similar genome-wide significance was not demonstrated in similar studies in cutaneous leishmaniasis (CL) (Fakiola et al., 2013). In VL, these studies highlighted the importance of antigen presenting cell function and regulation of IFN-y in host-parasite interaction (Fakiola et al., 2013; Blackwell et al., 2020). Interestingly, development of PKDL was linked to the decreased function of the interferon-gamma receptor 1 gene (*IFNGR1*), that was not found in VL. This was also found in skin biopsies from PKDL patients both from Sudan and India, with uniform low expression of IFN-y and *IFNGR1*, possibly explaining the persistence of parasites (Ansari et al., 2008; Farouk et al., 2010). Further studies are needed to explain differences within East-Africa and within the ISC, to describe the differences in PKDL rate, clinical presentation and interval between VL and PKDL.

Pharmacogenomics is the study of the response drug therapy in relation to variations of DNA and RNA characteristics; pharmacogenetics studies variations in DNA sequence in relation to drug response (Roden et al., 2019). This may be done by whole genome screening, whole exome screening or custom target sequencing microarrays (Mitropoulos et al., 2015). In Africa, as well as in Asia, genomic analyses have demonstrated that considerable genetic polymorphisms exist in the cytochrome P450 that may translate to differences in drug response (Mitropoulos et al., 2015).

In a more recent meta-analysis on the allele frequencies for 2 common liver enzymes in drug metabolism (CYP2D6 and CYP2C19), a mean risk of an abnormal CYP2D6 or CYP2C10 activity was found to be 36.4% and 61% respectively; there was major geographical variation: the range for abnormal CYP2D6 activity varied between 61% (Algeria) and 2.7% (Gambia), and for abnormal CYP2C19 between 80% (India) and 32% (Mexico) (Koopmans et al., 2021). This is important in the classification of individuals as slow, normal, rapid or ultra-rapid metabolizers. This means that in individuals who are not normal metabolizers, administration of a drug may cause (increased) toxicity or leads to ineffective treatment. For example, codeine is activated by CYP2D6 to its active metabolite morphine; in slow metabolizers this may lead to absence of a drug effect where as in rapid metabolizers it may lead to toxic and dangerous levels of morphine (Roden et al., 2019). Of the currently used anti-leishmania drugs, only pentamidine is metabolized by cytochrome P450 (Kip et al., 2018). Clearly, these concepts are also important for any co-administered drug in VL treatment.

The establishment of a biobank and pharmacogenetics database as for African population facilitates genomic research in diverse populations (Matimba et al., 2008). Fortunately, the cost of sequencing and genotyping is decreasing due to technological advances and national genomics research and policies are within the reach of governments in low-and middle income countries (Tekola-Ayele and Rotimi, 2015). This area of research has been neglected so far, despite inter- and intraregional differences in response to anti-leishmanial treatment.

In addition, more than 300 proteins are involved in ADME (absorption, distribution, metabolism and excretion) of drugs; there is insufficient information on the (variation) of genomic architecture underlying this (Chaudhry et al., 2016). This needs to be further explored in each population affected by leishmaniasis, to predict efficacy and/or toxicity of (new) drugs (Matimba et al., 2008; Baker et al., 2017; Kip et al., 2018). In case of severe toxicity, pharmaco-economics (the economic evaluation of drug administration) may be useful in defining cost-effectiveness of screening prior to administering treatment. For example, testing for glucose-6-phosphate dehydrogenase deficiency (G-6-PD) to avoid drug-induced hemolytic anemia such as caused by primaquine administration in the treatment of *P. vivax* malaria (Mitropoulos et al., 2015).

##### 3.1.1.1 Sex and Gender

Sex refers to biological differences while gender refers to social differences, relating to specific behavior, activities and roles of men and women in society (Mauvais-Jarvis et al., 2020).

The male to female ratio of VL cases in the Indian subcontinent reported by health facilities (1.40 [95% 1.37-1.43]) is similar to the risk ratio of incident VL during door-to-door screening (risk ratio 1.27 [95% CI 1.08-1.51]). Population-based studies showed that males were more likely to be seropositive in the DAT at baseline and more likely to develop VL or remain seropositive. These differences were not found below the age of 15 years (Cloots et al., 2020). Other studies showed that male sex was a risk factor for death or VL relapse, especially after puberty. The role of sex hormones has been established in animal experiments, and hormonal intervention has been suggested. Other factors may be involved such as the causative parasite as male risk is increased in *L. donovani* infection, but not in *L. tropica* (Lockard et al., 2019).

The influence of sex hormones on modulation of the immunity is poorly investigated; this may affect differences in outcome between males, and females, as well as non-pregnant and pregnant females. Males are more commonly affected with more serious clinical features, and it thought this is true despite better access to health care and more exposure to sand flies (Snider et al., 2009).

##### 3.1.1.2 Co-Infection

Protective immune responses in leishmaniasis are required for strong and long lasting immunity by persistent parasites through concomitant immunity (Mandell and Beverley, 2017). In patients who are cured, persistent replication takes place, mostly in activated antigen-presenting cells. In *L. major* infection, these are at the site of infection and draining lymph nodes and stay there for the rest of the host’s life (Okwor and Uzonna, 2008). It follows that any interference either by an infective agent or drug treatment may result in reactivation and clinical recurrence.

VL may occur with co-infections of other common (tropical) conditions, of which HIV infection and malaria are most common.

Immunosuppression is a risk factor for VL; this includes HIV infection, the use of immunosuppressant drugs (including steroids), haematological malignancies and patients with transplants (van Griensven et al., 2014). HIV co-infection causes increased susceptibility of infection, more severe disease, and diagnostic and therapeutic problems. This was also described for PKDL (El Hassan et al., 2013; Zijlstra, 2014). In IV drug addicts, a second mode of transmission cycle may exist between humans through exchange of contaminated needles. Conversely, *Leishmania* infection influences the clinical course of HIV infection (van Griensven et al., 2014). Tuberculosis may occur in VL-HIV co-infection influencing outcome (van Griensven et al., 2018). PKDL and VL with concomitant skin lesions are common and more severe in HIV co-infection (Zijlstra, 2014). Similarly, malnutrition may aggravate (HIV-related) immunosuppression, but its role is unclear as weight loss as a clinical feature may be caused by VL (Akuffo et al., 2018).

A different mechanism was described in the treatment of hepatitis C with Direct Acting Antivirals (DAA) that downregulate interferon and interferon receptors, thus leading to re-activation of previous leishmania infection (Colomba et al., 2019).

Helminth infection affects up to one-third of the global population and cause chronic infection with downregulation of the pro-inflammatory immune response an enhanced Th2 response and repair mechanisms. Chronic helminth infection suppresses antibacterial, antiviral and antiprotozoal immunity, leading to increased susceptibility and attenuated immunopathology (McSorley and Maizels, 2012). This may be modulated by the excretory and secretory products (the secretome) (Ditgen et al., 2014). The effect on susceptibility and severity of a concomitant disease may be aggravated by helminth-induced anemia and malnutrition (Ditgen et al., 2014). Currently, there is no evidence that helminth infection predisposes to more severe disease (in VL) or treatment failure of stibogluconate (in CL) (Tajebe et al., 2017; Martinez et al., 2020). The role of helminth infection as a trigger to develop PKDL has not been studied. Gut bacteria were also shown to be possibly implicated as VL patients showed dysbiosis of gut flora compared to healthy controls (Blackwell et al., 2020).

##### 3.1.1.3 Unexplored Co-Infection

The role of co-infection remains largely unexplored; measles may be taken as an example that continues to be a world-wide problem despite the availability of a vaccine. While natural infection induces a strong cellular and humoral immune response, after cure a transient immune suppression with increased morbidity and mortality may occur (Griffin, 2010).

Recently, it was shown that measles virus infection has an impact on circulating lymphocyte subsets, with memory T and B cells being most severely affected, causing loss of immunological memory and renewed to susceptibility to pathogens (Mina et al., 2019). Childhood vaccinations such as for measles are associated with reduced risk of measles (Lima et al., 2018). It has been suggested that measles could lead to breakdown of immunity; in a case report recurrence of VL after PKDL occurred after measles infection. Similar co-infections that may reduce immunity are malaria, tuberculosis and Epstein-Barr virus infection (Louzir et al., 1993; Nandy et al., 1998). There are no formal studies that examine the effect of measles or other common viral infections such as hepatitis B and hepatitis C on (recurrence of) VL or the occurrence of PKDL after seemingly successful treatment of VL. Concomitant malaria in VL was found to be associated with a more severe clinical picture of VL, including severe anemia, and increased mortality in Sudan (van den Bogaart et al., 2013).

##### 3.1.1.4 Genetic Determinants of Response to Infection

Transcriptomics describes the immunological response to infection by assessing the gene expression (‘signature’) in the interaction between host and pathogen. Using whole blood transcriptional profiling, the gene expression profile of active VL was demonstrated to be different compared with healthy controls, but this was not found in asymptomatic infection and healthy controls. In addition, support was found for more effective cure at 30 days post treatment for single dose AmBisome vs. multi-dose conventional amphotericin B for 30 days (Fakiola et al., 2019). In a recent study, an analysis of the whole blood transcriptome in HIV-VL co-infected patients identified 4 genes that accurately determined the treatment outcome (Adriaensen et al., 2020). This is a promising tool that needs further exploration, particularly to the correlation with actual immunity and immune parameters thereof in various clinical settings and endemic regions.

#### 3.1.2 The Parasite

Identification of the causative parasite in each clinical and epidemiological setting is crucial. Issues in taxonomy and species typing have been thoroughly reviewed (Van der Auwera and Dujardin, 2015).

While *L. donovani* causes VL both in Asia and Africa, molecular analysis of the parasite shows that there are three clusters: one East African cluster (Sudan, Ethiopia), a second with mixed Indian and Kenyan isolates and a third with *L. infantum* (Kuhls et al., 2007; Lukes et al., 2007; Jaber et al., 2018). It is not clear to what extent these differences explain different PKDL rates, performance of serological (rapid) diagnostic tests or response to treatment, and these should be understood in relation to the host immune response.

Molecular typing of the parasite has provided insight in the differences in epidemiological, clinical features within east Africa where *L. donovani* is the common causative agent in the whole region. In Sudan/North Ethiopia (NE group), the sand fly is *P. orientalis*, the PKDL rate is high and the response to paromomycin <67%, while in contrast, in South Ethiopia (SE group), the sand fly is *P. martini* and the PKDL rate is low, with better cure rates for paromomycin (>87%) (Hailu et al., 2010; Zackay et al., 2018). Whole genome analysis has demonstrated a strong correlation between one single nucleotide polymorphisms (SNP) in *L. donovani* and NE and SE groups, with genes involved in parasite viability and resistance to drugs (Zackay et al., 2018).

The methodology used is important; while southern blotting using a DNA probe to the *L. donovani* 28s rRNA gene did not show difference between NE and SE, amplification fragment length polymorphisms (AFLP) grouped the strains according to geographical origin with further subdivision in subpopulations by zymodeme, geography and year of isolation, but not by clinical features. Interestingly, skin isolates had fewer polymorphic AFLP fragments than VL strains, but strains of most VL and PKDL patients grouped together, suggesting that unidentified genetic factors may underlie development of PKDL (Jaber et al., 2018). Another study found polymorphism in the HASPB repeat region (also known as k26) of east African *L. donovani* strains (north Ethiopia, Sudan and Kenya) which could influence the performance of a diagnostic test or a vaccine based on k26 (Zackay et al., 2013).

In Brazil, natural resistance of *L. infantum* to miltefosine was demonstrated in VL patients who failed to respond to treatment and was linked to a Miltefosine Sensitivity Locus (MSL) by the use of Genome-Wise Association Study analysis of SNPs, gene and chromosome copy number variations (Carnielli et al., 2018; Carnielli et al., 2019).

In the ISC, *L. donovani* causes VL while in Sri Lanka, it also causes CL. A recent study suggested that SNPs and variable gene expression causes differences in tropism (Samarasinghe et al., 2018). Other genomic studies have demonstrated that *L. donovani* strains may form a genetically distinct population that are the basis of resistance to stibogluconate, rather than SNPs (Samarasinghe et al., 2018).

Also, in the ISC, there is no convincing evidence that strains causing VL and PKDL differ, but there are few data on paired strains and most studies are cross sectional. In addition, only minor differences in strains from VL and PKDL have been demonstrated so far, except for drug sensitivity (Dey and Singh, 2007; Subba Raju et al., 2008; Mishra et al., 2013). However, some proteins are upregulated such as Gp63 and PSA, that may alter the parasite and promote accumulation in the skin (Salotra et al., 2006). Parasite strains from Sudan examined by PCR single strand conformation polymorphism did not show correlation with clinical manifestations (Bermudi et al., 2020). VL and PKDL strains clustered in the same branches of the tree examined by multi-locus microsatellite typing (MLMT) or multi-locus sequence typing (MLST) (Alkhaldy et al., 2020; Bolia et al., 2020).

##### 3.1.2.1 Co-Infection With Endosymbionts

*Leptomonas seymouri* is an opportunistic parasite that is assumed non-pathogenic to humans but may be an opportunistic pathogen in HIV infection. It has also been demonstrated in VL and PKDL patients and HIV infected patients with diffuse cutaneous leishmaniasis (DCL) (Dedet et al., 1995; Ghosh et al., 2012). Co-infection was demonstrated in 13.8% of patients with VL/PKDL caused by *L. donovani* in India. The implications hereof are not clear, including a role in antimony unresponsiveness to stibogluconate (Ghosh et al., 2012). Recently, it was also demonstrated in cutaneous leishmaniasis caused by *L. donovani* in India, suggesting a role in pathogenesis as *L. donovani* generally causes visceral disease. It has also been demonstrated in sand flies. There is similarity in the genome of leptomonas and *Leishmania*. The biology and role of leptomonas as a co-infecting parasite or a hybrid of *Leishmania* needs further investigation (Thakur et al., 2020).

Species of *Leishmania* may show the presence of Leishmania RNA virus (LRV), member of the *Totiviridae*, that has been associated with treatment failure and relapse in CL caused by *L. braziliensis* and *L. guyanensis* infection (Adaui et al., 2016; Macedo et al., 2016). Its role remains controversial; a recent study in CL caused by *L. guyanensis* did not show a relationship between treatment failure to pentamidine treatment or the presence or genotype of LRV (Ginouves et al., 2021). In another study, it has been associated with development of Diffuse Cutaneous Leishmaniasis (DCL) (Rossi and Fasel, 2018). LRV viral DNA interacts with the Toll-like receptor 3 (TLR3) on the macrophage and triggers the production of pro-inflammatory cytokines including interferon-β and thus confers a selective advantage to *leishmania*, include its retention and capacity to metastasize (Grybchuk et al., 2020). Its action is through production of type I interferons suggesting that other viruses may have a similar effect (Rossi et al., 2017). A first non-LRV RNA Leishmania-infecting leishbunyavirus has been described (Grybchuk et al., 2020).

#### 3.1.3 The Sand Fly Vector and Transmission

Major progress has been made to describe the development of the leishmania parasite in the sand fly and factors determining transmission during a sand fly bite. Genetic exchange between *Leishmania* parasites occurs within the sand fly contributing to phenotypic diversity that influences resistance, virulence and tropism (Akopyants et al., 2009). In addition to leishmania parasites, sand flies may be infected and transmit viruses such as phleboviruses. Preliminary reports suggest that infection by *Leishmania* and (phlebo) viruses is not compatible in the sand fly midgut and may affect leishmanial development; this could be further studied in the context of mode of *Leishmania* control (Telleria et al., 2018). Similarly, *Leishmania* development in the sand fly midgut may be inhibited by other microbiota such as bacteria and fungi that are co-egested with *Leishmania* during the blood meal and offer possibilities for paratransgenic or biological control as a prophylactic or therapeutic option (Campolina et al., 2020; Karmakar et al., 2021). In the mouse model these microorganisms may facilitate the establishment of *Leishmania* infection by activating the neutrophil inflammasome with production of IL-1b (Dey et al., 2018). Sand fly saliva is equally co-egested with the *Leishmania* parasites during a bloodmeal and has anti-hemostatic, anti-inflammatory, and immunomodulatory properties. In the naive host sand fly saliva promotes *Leishmania* infection with larger numbers of parasites and larger lesions. Repeated exposures result in protection as the host is immunized by salivary proteins. This potential is supported as bites from non-infected sand flies outnumber those from infected sand flies (Karmakar et al., 2021). These antigens may be exploited in vaccine development, whereas anti-saliva antibodies may be used in epidemiological studies (Lestinova et al., 2017). The role of saliva from vectors other than sand flies merits further study (Karmakar et al., 2021).

### 3.2 Impact of New Genomic and Non-Genomic Tools on Insights to Precision and Identification of Priorities

#### 3.2.1 Diagnosis

Current pitfalls in diagnosis include the use of highly sensitive diagnosis by molecular tools for demonstration and typing of the parasite in various tissues including peripheral blood, replacing classical microscopy and culture. Second, serological tests have poor specificity as antibodies may persist from previous leishmanial infection. Third, molecular parasitological methods may not be as useful as test of cure (biomarker) as immunological parameters that reflect the developing immune response rather than disappearance of the parasite (Salotra et al., 2001; Antinori et al., 2007; Boelaert et al., 2014; Sundar and Singh, 2018; Zijlstra, 2019).

##### 3.2.1.1 Demonstration of Parasites

Demonstration of parasites by microscopy is still considered the gold standard in diagnosis of VL and PKDL in many endemic areas, and the preferred method in patient care, particularly in Africa, or in research projects. Microscopy requires training and a working microscope; the parasite load may be quantified, e.g. in a spleen aspirate (Sokal, 1975). All patients are treated with the same regimens independent of the parasite load; precision to the individual level has not been studied. Digital imaging and reading of slides is under development and allows reading with adjustment of focus and magnification, and may be used for quality assurance. In malaria, for example, a timed tally counter for reading thick blood films, was found to provide richer, more accurate data, and less time consuming (Nuel and Garcia, 2021). So far, no studies have been published in leishmaniasis.

Antigen tests differ in sensitivity and specificity with lowest and highest sensitivities are reported from Nepal/Tunisia and Europe/Middle East respectively (p < 0.05), and the lowest and highest rates of specificity were reported from Sudan and America/Middle East, respectively (Fararouei et al., 2018).

Microscopy and culture are gradually superseded by molecular tools, such as PCR; a pan Leishmania PCR may be done or a species-specific PCR particularly if a certain subspecies is suspected. Subgroups may be identified by various molecular methods (Van der Auwera and Dujardin, 2015). PCR is usually done on aspirates of lymph node, bone marrow or spleen aspirate, with increasing sensitivity reaching 95-97% in a spleen aspirate (Zijlstra and el-Hassan, 2001). In HIV-VL infected patients, PCR in blood may be used (Antinori et al., 2007). Antigen tests should also be carefully evaluated against the superior sensitivity of PCR based diagnosis and test of cure.

Challenges include the use of PCR in the field; using modifications to classical PCR such as LAMP and recombinase PCR can be done in the suit-case field laboratory (Verma et al., 2013; Mondal et al., 2016). For differential diagnosis, a multiplex PCR may be developed; this would be a simultaneous test for malaria, schistosomiasis, or brucellosis, or co-infection with HIV according to the local epidemiology; this requires regional precision.

Parasite detection by PCR as a test of cure indicates disappearance of the parasite but not necessarily a protective immune response. qPCR offers quantification of the parasite load and may be useful as a biomarker (Wu et al., 2020). Recently, qPCR in blood of non-HIV infected VL patients 2 months after start of treatment was shown to correlate well with tissue parasite load and predict treatment response (relapse) in East Africa (Verrest et al., 2021).

##### 3.2.1.2 Antibody Tests

In VL, a strong humoral response exists as evidenced by polyclonal gamma-globulinaemia; this consists mainly of IgG antileishmanial antibodies that may be used in serodiagnosis, reflecting largely the Th2 immunological response.

Multiple serological antibody tests have been designed and studied in the past decades; these mainly include the direct agglutination test (DAT), rK39 ELISA and IFAT. The sensitivity is satisfactory, but a major problem exists with specificity. While this may be influenced by cross-reaction with other antigens, in most cases antibodies that are detected may result from previous exposure to *Leishmania* (clinical or subclinical) as these antibodies may persist for many years (Khalil et al., 2013). Often sensitivity and specificity are determined by testing stored sera, using healthy endemic controls or sea from patients with other diseases such as malaria. It is however essential to evaluate a diagnostic test in a prospective study, with proper follow-up of those tested positive without clinical features, to establish the outcome and the measure positive and negative predictive value (PPV and NPV, respectively). It should be noted that the PPV may vary according to prevalence of the disease and perform better when transmission is high, such as during an outbreak.

The rK39 rapid diagnostic tests (RDT) is the only RDT that has been successfully developed in VL; it was found 90% sensitive and 100% specific in India, during a time of high prevalence of VL, and the test is used in the KAEP in the ISC (Kumar et al., 2001). However, false positive results may occur in malaria, tuberculosis, liver cirrhosis and chronic myeloid leukaemia (Topno et al., 2020). In a Cochrane review, the rK39 immunochromatographic test (RDT) showed overall sensitivity in the ISC was 91.9% (95% CI 84.8-96.5) vs 85.3% (95% CI 90.0-99.5%) in East Africa. In East Africa, the sensitivity was lower possibly because of lower antibody production below the cut-off point for detection that may be a function of parasite virulence (Zijlstra et al., 1998; Boelaert et al., 2014). This issue shows the precision needed at regional level. All other tests lack accuracy, validation, or both (Boelaert et al., 2014). A re-evaluation of the performance of the rK39 RDT (positive predictive value) in the KAEP may be useful, as the prevalence of VL has dropped dramatically. (www.who.int/en/newsroom/factsheets/detail/leishmaniasis). Other antibody tests are based on rK9 and rK26 antigens; these were shown to have lower sensitivity than rK39 in India (Mohapatra et al., 2010). Studies in other regions are awaited.

Targeting multiple antigens may be useful to increase test performance. Recently, a monoclonal antibody-based multiplex capture ELISA with five different monoclonals to leishmania proteins was found to have improved sensitivity of ≥ 93% and 100% specificity in urine samples from Brazil and Kenya (Abeijon et al., 2020).

##### 3.2.1.3 Immunological Tests

The cellular immune responses in VL and PKDL have been recently reviewed (Zijlstra, 2016). For convenience, the immune response is described in terms of a Th1 and Th2 response; while in the animal model this dichotomy is genetically determined, this has not been demonstrated in humans where markers of both Th1 and Th2 response co-exist in VL and vary during treatment or in the natural history (Zijlstra, 2016).

In VL, the Th2 response is characterized by the absence of a cellular immune response against *Leishmania* parasites: peripheral blood mononuclear cells (PBMC) do not proliferate in response to stimulation with leishmanial parasites; *in vivo* the leishmanin skin test (LST) is typically negative. While there is a predominant role for anti-inflammatory cytokines (Th2 response) such as IL-10 and TGF-ß, also high levels of interferon-γ (INF-γ) can be demonstrated that is typically associated with healing (Th1 response) suggesting that there is no Th1 defect in active VL. This shows the complexity of the immune response that cannot be captured in a Th1 and Th2 dichotomy alone (Faleiro et al., 2014). Various other mediators need to be described according to disease stage, severity and outcome (Brodskyn and Kamhawi, 2018).

Analysis of cytokines responses requires an advanced laboratory. Recently, a novel point-of-care device was presented that directly monitored a panel of five cytokine markers that may reflect the host’s immune response to sepsis. It used a sensor disposable cartridge attached to an electrochemical reader. This could have great potential in VL and PKDL as a marker of infection and as a biomarker to monitor the response to treatment (Tanak et al., 2021).

Cytokine panels may identify crucial levels or a ratio of key anti-inflammatory and pro-inflammatory cytokines that correlate with diagnosis, improvement during treatment, cure in VL as well as prediction of PKDL. Such panels have the optimal potential to also serve as a biomarker; it should be tailored to precision at the regional, or intra-regional level.

New approaches that measure genetic determinants of infection such as a gene signature in a transcriptomics approach are promising (Fakiola et al., 2019; Adriaensen et al., 2020).

The *Leishmanin skin test* (LST) (commonly called the Montenegro test in Latin America) measures type IV cell-mediated immunity. Intracutaneous injection of killed *leishmania* parasites induces induration that is measured after 48-72 hours (Sokal, 1975). Normally, the LST is negative in VL, may be positive or negative in PKDL and becomes positive after cure (Zijlstra and el-Hassan, 1993). There is no consensus about a cut-off size of induration for a positive test; while classically 5 mm is used, others consider any induration a positive result.

The LST is easy to use and may obviate the need for testing immune responses in the peripheral blood. It identifies susceptible individuals as these are LST negative; a positive LST indicates previous exposure (clinical or subclinical) and is generally considered a parameter of a Th1 response and an indication of immunity (Gramiccia et al., 1990; Zijlstra and el-Hassan, 1993; Schaefer et al., 1994). It is however not clear if a positive LST result indicates life-long protection and whether repeated exposure is required. In immunosuppression a positive test may become negative which correlates with increased chance of re-infection or recurrence of a previous infection.

The LST is a powerful epidemiological tool. Its use may be demonstrated in control programs such as the KAEP in the ISC to monitor herd immunity once transmission is under control; similarly a study in Sudan demonstrated that a positive LST was associated with protection for VL (Zijlstra and el-Hassan, 2001). Alternatively, it is useful to screen for non-immune individuals in a prophylactic vaccine trial (Khalil et al., 2000; Regional Strategic Framework for Elimination of VL From SEA Region (2005-2015)). In other epidemiological cross sectional studies, LST positivity rates were not consistent and it is not certain if this is due to lack of boosting by repeated exposure to sand fly bites or to the poor performance of the LST antigen used (Bern et al., 2006).

Currently there is no LST antigen available because of issues, among other, in Good Manufacturing Practice (GMP). *L. major* and *L. infantum* antigen have been used most and were satisfactory in detecting previous *L. donovani* infection (Zijlstra and el-Hassan, 1993). *L. amazonensis* antigen has also been used with better sensitivity than *L. infantum* (Bern et al., 2006). The LST, provided it is manufactured by GMP, may still be a useful and powerful epidemiological tool in control programs, to assess immunity in the individual and in the population; it will need to be assessed for accuracy in different populations. There is an urgent need for a new LST manufactured according to GMP, with *L. donovani* antigen; research efforts are on-going. (https://www.ghitfund.org/investment/portfoliodetail/detail/159)

Metagenomic next-generation sequencing (mNGS) may offer opportunities for screening for unexpected co-infection. It complements other (routinely used) molecular based diagnostics as it aims to identify any nucleic acid in the samples in an unbiased way and thus may identify organisms that not suspected and otherwise cannot be cultured. It also provides information on subspecies and antimicrobial susceptibility. Disadvantages include contamination and lower sensitivity for organisms with small genomes or present in low quantities (Ko et al., 2019; Li et al., 2021). While this technology has the advantage of diagnosing co-infections, the significance of finding a second microbe should be carefully assessed as to its clinical relevance. It has been used in leishmaniasis on e.g. bone marrow aspirate and trephine for identification and speciation of Leishmania (Williams et al., 2020). This option may be further explored in the differential diagnosis of infectious diseases, using more accessible samples such as blood in VL or a skin slit smear in PKDL. New portable and affordable devices may be introduced in resource-limited settings (Lu et al., 2016). Other applications include a meta-taxonomic analysis of gut microflora using 18S rRNA gene sequencing that showed dysbiosis of bacterial diversity in the gut of VL patients compared with healthy controls; this could be explored further (Blackwell et al., 2020). Metagenomic studies may be used to address unsolved issues in co-infection and the effect thereof in immune responses, including the late occurrence of PKDL in the ISC.

Nanotechnology (nanodiagnostics) is based on biomedical detection systems that do not require a read-out tool such as a gel, PCR or culture; they are rapid and seem cost-effective for use in the field. In VL, the potential for use in an elimination program was suggested because of superior sensitivity for early detection and simultaneous detection of drug resistance (Singh et al., 2019; Gedda et al., 2021). Theragnostics combines diagnostics and therapeutics using nanotechnology and holds promise for accurate monitoring of infection in the field. Rather than measuring gene polymorphism as in pharmacogenomics, it is more flexible and may use combined information from genomics, proteomics and metabolomics (Ozdemir et al., 2006).

#### 3.2.2 Treatment

##### 3.2.2.1 Treatment of VL

Developments in drug treatment in the past decades have focused on drug resistance (sodium stibogluconate, SSG), the first oral drug (miltefosine), combination therapy and targeted drug delivery (AmBisome). All are primarily aimed on elimination or reduction of parasites leading to cure and preventing relapse. Pitfalls in treatment of VL were summarized in a recent review (Alves et al., 2018). These include regional and intra-regional differences in cure rates of anti-leishmania drugs, resistance, the need for combination treatment, risk of PKDL and adequate dosing guided by pharmacokinetic studies.

There are important regional differences. In some these differences seem related to the drug used and host; AmBisome and miltefosine have lower cure rates in Africa compared with the ISC (Musa et al., 2010; Wasunna et al., 2016). This has led to the adoption of short course SSG +paromomycin combination therapy for 17 days as the standard treatment; this combination was found to be non-inferior was demonstrated to 30 days of SSG, thus decreasing SSG related toxicity (Musa et al., 2012). In the KAEP in the ISC single dose AmBisome is one of the pillars to success (WHO, 2011; Musa et al., 2012).; second line treatment may be with AmBisome combined with miltefosine and paromomycin, respectively (Sundar et al., 2011). While SSG is no longer used in the ISC because of resistance, this is not reported from East-Africa. The poor performance of miltefosine in VL caused by in Brazil was linked to the parasite (*L. infantum*) (Carnielli et al., 2018).

Miltefosine was found to fail in the treatment of VL in children as a result of variable drug exposure. In a recent trial on treatment of children with VL, linear dosing was replaced with allometric dosing with less variable exposure and increased efficacy, thus providing precision to group level (Mbui et al., 2019).

The treatment of VL-HIV co-infection was recently studied; a combination of high dose AmBisome (30 mg/kg total dose) and miltefosine 100 mg daily for 30 days was found superior to monotherapy AmBisome (40 mg/kg total dose), showing that precision may be applied to the group level (those who are HIV infected), while confirmation is awaited from other studies in the ICS (Diro et al., 2019).

##### 3.2.2.2 Risk of PKDL

Drug treatment of VL is an important factor in the incidence of PKDL. In a follow-up study in Bangladesh of VL patients treated with monotherapy (SSG, miltefosine, paromomycin, single dose AmBisome [SDA], multidose Ambisome), or combination therapy (AmBisome + miltefosine, AmBisome + paromomycin, miltefosine + paromomycin), it appeared that SSG and multidose AmBisome had significant lower PKDL and relapse incidence rates than the other regimens (Mondal et al., 2019). In a similar study from India, SDA had a lower PKDL risk than miltefosine + paromomycin, with higher risk in females and children < 12 years. Children also had a higher risk of relapse (Goyal et al., 2020).

In Africa, PKDL incidence was found to be high in those treated with poor drug treatment (low dose, incomplete courses, poor quality SSG), and in young children. Treatment with paromomycin lead to higher PKDL rate in those treated with low dose of longer duration (Hailu et al., 2010).

Recently, the clinical pharmacokinetics of systemically administered anti-leishmania drugs was reviewed (Kip et al., 2018). While PK data have been described for pentavalent antimony, miltefosine and paromomycin, there are no data on AmBisome and pentamidine. Only miltefosine has been studied to define target exposure levels to effect. Few data exist on paediatric populations and female patients who are pregnant or breastfeeding. Here, precision to the individual (group) level is needed. In HIV-VL co-infection, drug-drug interactions, renal clearance, and protein-binding should be taken into account; conversely, the PK of antiretroviral drugs has not been evaluated in VL (Kip et al., 2018). The importance of pharmacokinetics was recently demonstrated in HIV-VL co-infected patients where low drug exposure of AmBisome and miltefosine was found, possibly explaining the high relapse rate in this patient group (Kip et al., 2021).

##### 3.2.2.3 Vaccination and Immune Manipulation

Leishmanization is the oldest and best form of immunization so far. Inoculation with live virulent *L. major* parasites induces a lesion that fades after 6 months. It was abandoned since inoculation did not induce a lesion in everyone. As the vaccine contains live parasites, it is not stable and thus not practical.

The first vaccines used autoclaved whole parasite leishmania strains such as *L. major* or *L. mexicana*; later specific parasite antigens were used. This was followed by vaccines that used antigen delivery through a viral vector, or that used modification or deletion of genes. Various adjuvants and immune-enhancers have been used, including bacille Calmette-Guerin vaccine (BCG) (Zijlstra et al., 2020).

In the only prophylactic trial in VL, using an autoclaved *L. major* vaccine, no adequate protection for VL was found in Sudan (Khalil et al., 2000). The same vaccine was found to be efficacious in immuno-chemotherapy in patients with chronic PKDL, resulting in LST conversion and increased IFN-γ/IL-10 production (Musa et al., 2008).

In addition, research is on-going on an immunomodulator (CpG oligodeoxynucleotide) that stimulates plasmacytoid dendritic cells; it induces maturation of monocytes into mature dendritic cells and is a strong Th1 adjuvant. It is under development by DND*i* in collaboration with the US Food and Drug Administration. (www.dndi.org).

##### 3.2.2.4 Way Forward

The outcome of VL is determined by the balance between antigen-specific anti-inflammatory cytokine and pro-inflammatory responses that are determined by host genetic factors (Blackwell et al., 2020). Differences in drug responses should be understood in each noso-geographical region with regard to cure, relapse and PKDL rates after each VL drug regimen, in relation to population, parasite and sand fly characteristics. In this respect, it should be noted that not all these issues have been rigorously studied with comparable methodology in all regions. In each of these different outcomes, genetics and immunological factors play a role. In addition, as VL patients are immunocompromised, secondary infections and malnutrition may influence outcome.

Developing new drugs requires a lengthy and costly investment with an uncertain outcome. New candidate molecules are often being explored based on the killing potential of leishmanial parasites *in vitro*, while the immune response induced by drug treatment is the most important indicator for treatment success or failure. This is often not addressed or appreciated until well into the clinical stages of development, in phase II and III studies.

In addition, each drug may have intrinsic immune effects that have not been sufficiently described (Ansari et al., 2008; Mukhopadhyay et al., 2012; Ghosh et al., 2013). This was recently reported in detail for miltefosine that induces enhanced Th1 cytokine responses (Palic et al., 2019). This shows that drug development should be driven by outcome (immunity) rather than parasite killing ability alone. Sterile immunity does not exist and parasites persist under the control of the immune response. Parasite eradication may be detrimental to the host’s immunity and thus to herd immunity (Okwor and Uzonna, 2008; Mandell and Beverley, 2017).

AmBisome is the prototype drug for VL targeted drug delivery as it is phagocytosed by (infected) macrophages and accumulates in liver and spleen, with reduced renal and infusion-related toxicity compared to amphotericin de-oxylate. Nanotechnology (nanoparticle-based drug delivery) holds promise to further refine targeted drug delivery with the potential to decrease drug regimens, drug resistance, drug toxicity, and cost. Further research is on-going which should address nanotoxicity, biodistribution and post exposure bio-persistence, as well as theragnostics (Singh et al., 2019).

Randomized Clinical Trials (RCTs) provide precision but are precise for a subset of the population as even with randomization, participation is not uniform and do not take into account other health determinants such as social and demographic factors; these include women (pregnant or in child-bearing age) and children. It follows that all RCTs should be performed in each endemic region in all patient groups affected to address these factors. For this purpose, these should include a diagnostic study to detect co-infections, a study of biomarkers, a PK/PD assessment, pharmacogenomics, and analysis and genotyping of the circulating parasites. A study design in which patients are stratified according to parasite load and outcome may provide precision of treatment to the individual level. The recent study on allometric dosing of miltefosine in children emphasizes the precision needed in patient groups that are not primarily included in RCTs, such as children; the same would apply to (pregnant) women (Mbui et al., 2019).

A number of checkpoint molecules have been identified that provide opportunity for targeted intervention; these are present on CD4+ cells in VL patients, including CTLA-4 (CD152) and PD-1, which negatively regulate T-cells. CTLA-4 binds to costimulatory ligands B7-1 and B7-2; activation of CTLA-4 leads to increased levels of TGF-ß. PD-1 binds to PD-1 ligand 1 and blockade of either promotes parasite clearance and increased pro-inflammatory cytokine production in experimental VL (Kumar et al., 2017).

Population pharmacokinetic modelling offers insight in drugs with large variability in exposure and the use of allometric dosing; it has the advantage that sparse and heterogenic sampling may be used and this would be suitable for research in remote settings. For this, blood samples collected as dried blood spots are attractive. Special areas of interest are intra-cellular drug levels and drug levels in the skin vs. systemic levels (Kip et al., 2018).

Investment in drug development needs to be balanced, for example, against investing in vaccine development. Vaccination provides better precision than drug treatment as it aims directly at inducing a protective immune response. Pharmaco-economic assessment may indicate that investing in vaccine development may ultimately be more efficient and more cost-effective in control programs.

#### 3.2.3 PKDL

Current issues in PKDL include the inter- and intra-regional differences in pathophysiology, management and prevention (Zijlstra et al., 2003; Zijlstra, 2016; Moulik et al., 2018; Zijlstra, 2019).

##### 3.2.3.1 Pathophysiology

In Sudan, the natural history of VL and PKDL shows that 85% of PKDL cases self-cure, while 15% have persistent PKDL and require treatment. An inverted relationship exists between positive serology (DAT titer) and the LST positivity. Those who showed self-cure were more likely to have a positive LST and were less likely to have a positive DAT result. The opposite was found for those who developed persistent PKDL (Musa et al., 2002). Treatment of PKDL in Africa has been poorly studied; regimens include SSG + PM for 17 days or AmBisome for 10 days (Musa et al., 2005; Younis et al., 2016). Other than in Sudan, where PKDL occurs in the context of the developing immune response after seemingly effective treatment of VL, in the ISC the role of an intercurrent infection should be explored; this could be targeted to suspected microbes such as measles or malaria; alternatively, a metagenomic approach may help to identify thus far undetected concomitant microbes.

PKDL patients are different from VL patients as PKDL occurs at a younger age and PKDL is limited to the skin without systemic disease; the patients are on average healthy, not malnourished, and normally do not have anaemia or low albumin levels (Zijlstra et al., 2003). Also here the developing immune response determines the clinical differences and precision in treatment is needed within the spectrum of *L. donovani* infection based on PK/PD studies. Paired VL and PKDL strains from the same patients and strains from VL patients who do not develop PKDL should be analyzed to explore if genetic factors in parasites underlie development of PKDL.

##### 3.2.3.2 Management

Diagnosis of PKDL requires parasitological confirmation as the differential diagnosis may be difficult and in the ISC all patients are treated with miltefosine for 3 months. (q)PCR on a slit smear is more sensitive than microscopy (Verma et al., 2013). Recently, a microbiopsy device was found to accurately quantify the parasite load in the skin in PKDL (Cloots et al., 2021). The extent of the rash and at diagnosis and during treatment may be described clinically by grading, using a maniken, clinical photography or a combination of a clinical and parasitological score. There are no reliable parasitological, laboratory or immune biomarkers (Zijlstra, 2019). Molecular diagnosis may be done in the field using the mobile suitcase laboratory (Mondal et al., 2016). Novel 3-dimensional optical scanning is a novel tool to measure PKDL lesions with great accuracy with regard to diameter, surface and volume as was demonstrated from Sudan. It is suitable for use under field conditions (Zijlstra et al., 2020).

Diagnosis at field level should be further developed with regard to include the use of multiplex PCR panels in differential diagnosis to detect other infectious causes of a similar skin rash, such as leprosy and fungal infections (e.g. tinea). The use of the field laboratory may be expanded. The use of tele-medicine, artificial intelligence and diagnostic algorithms may be explored further to include all infectious and non-infectious differential diagnoses. A teaching course for health workers, a picture atlas and guidelines for management have been developed (WHO, 2012; WHO, 2013; WHO, 2017).

Treatment with anti-leishmanial drugs should be with extremely safe and effective drugs as PKDL usually causes limited morbidity without mortality. Regimens should be short course, oral administration allowing treatment on an out-patient basis. Group precision is needed as PKDL patients, in contrast to VL, are not ill and are not malnourished and PK/PD data are required. In addition, drug levels in the skin need to be studied *vis-a-vis* systemic drug levels, for each drug used, including intrinsic immune effects. This is currently being studied in drug trials in East-Africa and the ISC.

In the ISC all patients are treated with miltefosine for 3 months. A particular problem occurs in the evaluation of cure in macular PKDL as the repigmentation may take a long time and continues after parasitological and immunological cure (Verma et al., 2015; Zijlstra, 2016; Moulik et al., 2018). qPCR was useful to monitor treatment response in patients treated with miltefosine who had a good clinical and parasitological response while in patients treated with AmBisome the response was unsatisfactory as measured by qPCR (Moulik et al., 2018). Miltefosine resistance has been reported (Ramesh et al., 2015).

A new treatment modality to be explored is the use of biologicals. Blockade of PD-1 or PD-1 ligand may promote parasite clearance and increased pro-inflammatory cytokine production as was shown in experimental VL. In PKDL in the ISC, the presence of PD- 1 was demonstrated in dermal lesions by increased mRNA expression of PD-1 and by immunohistochemistry, and in the peripheral blood, while this decreased after treatment and was not demonstrated in healthy controls (Mukherjee et al., 2019). From a clinical point of view, an immune block seems to exist in PKDL in the ISC with no tendency to self-heal, in contrast to the experience in Africa. The use of biologicals in PKDL (and VL) warrants further investigation, for efficacy and safety, and cost-effectiveness.

Biomarkers would include cytokine ratios that could be used as a better, earlier and more direct reflection of the pathophysiology compared to parasitological tools; for example the ratio of a pro-inflammatory cytokine such TNF-α and IL-1 vs. an anti-inflammatory cytokine such as IL-10 and TGF-β could be measured before during and after treatment, as a sole cytokines or a part of a cytokine panel. The use of 3-dimensional scanning may be also explored further in diagnosis and as a biomarker in each of the main types (macular and papulonodular). These two modalities should provide precision at the regional level as the differential diagnosis may differ.

Immunochemotherapy may be second line treatment in chronic or refractory cases; the ChAd63-KH vaccine seems a suitable candidate to further build on previous experience with the autoclaved *L. major* vaccine (Younis et al., 2021).

##### 3.2.3.3 Prevention

Prevention is of paramount importance and seems within reach. Given the increasing understanding of the immune responses, immunological manipulation is attractive to prevent PKDL, and may be combined with drug treatment for VL to enhance or accelerate the immune response, leading to definite cure with reduced risk of VL relapse and development of PKDL. Given the differences in epidemiology and pathophysiology PKDL, more (intra-)regional precision is needed.

Infectivity of PKDL to sand flies should have priority as this is likely to be the driving force in transmission. A complete understanding is needed as to which patients contribute most and at which stage of PKDL, both in terms of numbers (quantity) as in terms of quality (macular vs. polymorphic; early vs. late, limited vs. generalized, etc.). While major progress has been made in infectivity studies from Bangladesh and India, no conclusive studies data exist from East Africa that have used modern tools such as PCR in large numbers of patients (Mondal et al., 2018).

#### 3.2.4 Asymptomatic Infection

Current pitfalls in asymptomatic infection include definition, pathophysiology, regional differences, progress to VL and infectivity (Zijlstra et al., 1994; Saha et al., 2017; Sundar and Singh, 2018; Das et al., 2020; Singh et al., 2021).

##### 3.2.4.1 Definition

After infection by a sand fly bite, not all individuals develop clinical VL. Theoretically, three groups may be identified: those who do not have any sign of infection; these remain undetected. The second group are those in whom (conversion in) a positive serological or immunological test is found, but are healthy; these are called asymptomatics. While most individuals remain asymptomatic, a small number develop clinical disease over time.

The third group develop clinical VL, as demonstrated by the presence of parasites or another diagnostic test.

There is no uniform definition of asymptomatic infection. Most studies would advocate the use of a serological test such as the DAT or rK39 ELISA; in longitudinal studies conversion from negative to a positive test would provide strong evidence of a recent infection. Other studies use the LST as an additional marker; here the presence of (conversion in) the LST would also provide evidence for infection leading to immunity. In HIV infected patients infected with *L. infantum*, cytokine release assays were shown to be useful tools (Ibarra-Meneses et al., 2017; Botana et al., 2019). The ratio of clinical VL to asymptomatic to varies from 1:9 in India and Nepal, while in Bangladesh this was 1:4. In contrast, in a study in Sudan, in the very beginning of an outbreak after a long disease free interval, clinical infection outnumbered asymptomatic infection by 2.4:1, suggesting low herd immunity (Zijlstra et al., 1994; Bern and Chowdhury, 2006; Ostyn et al., 2011).

There is a need for a more precise definition of asymptomatic infection in each noso-geographic region to understand its importance in transmission. This would take into account factors relating to the host, parasite and sand fly. Studies using molecular tools, immune markers and genetic studies are needed to further describe the importance of asymptomatic infection in terms accurately detection of infection, the risk of developing VL and the risk of transmission of progression to clinical VL, and the role in transmission.

##### 3.2.4.2 Pathophysiology

Clustering in households is well described and may be due transmission related factors such as the presence of VL or PKDL cases or the presence of sand flies and a potential animal reservoir. It is not certain if genetic predisposition in families may play a role (Bern et al., 2010).

It should be noted that the degree of immunity in asymptomatic infection cannot be quantified and this could vary over-time in the natural history, in the course of a concurrent infection, with age, in the course of an outbreak, or during on-going endemic transmission. For example, 32 of 42 asymptomatic individuals cleared the infection as demonstrated by qPCR within 6 months (Das et al., 2020).

##### 3.2.4.3 Progression to VL

The importance of asymptomatic infection lies in the presence of leishmania parasites and the possible progression to clinical VL; obviously these individuals need treatment. Markers that have been proposed include high antibody titres in serological tests (DAT, rK39 ELISA), ADA, IL-10, qPCR and LAMP (Chakravarty et al., 2019; Das et al., 2020).

Progression to VL is a considerable risk in HIV co-infection and a screen-and-treat strategy has been developed (van Griensven et al., 2014).

It may be useful to describe the developing immune response in terms of pro- and anti-inflammatory cytokine ratios such as between TNF-α or IL-10, and IFN-γ. Alternatively, measurement of adenosine deaminase (ADA) as an aspecific immune marker present in all tissues (isoenzyme ADA-1) or in monocytes macrophages (ADA-2) was suggested as a better marker than IL-10 to predict progression to VL (Vijayamahantesh et al., 2016).

Follow-up is essential in asymptomatic patients who are labelled on the basis of as having a positive serological test. It is important to detect those who progress to clinical VL; here molecular tools such as qPCR have proved indispensable. In a recent study in India, combined rK39 and DAT were find optimal to identify early asymptomatic infection; combining qPCR with serology detected 23.8% of asymptomatic cases that converted to clinical VL (Das et al., 2020). The clustering in households may be explored in association to genetic markers that have been linked to asymptomatic infection such as alleles of HLA-DRβ (Chakravarty et al., 2019).

##### 3.2.4.4 Infectivity

While for the asymptomatic individual progression to VL is obviously important, for the contribution of asymptomatics to transmission, xenodiagnostic studies are pivotal (Singh et al., 2020). For control efforts, the infectiousness of PKDL and asymptomatics as the main contenders, should be quantified in such experiments and balanced against their respective incidence.

Xenodiagnostic studies have been done in PKDL, and are on-going in asymptomatics in Bangladesh and India (Molina et al., 2017). A recent report from India did not provide support for a significant role of asymptomatics in transmission (Singh et al., 2021). In East Africa, xenodiagnostic studies have shown that 3.2% of the most infected people were responsible for infection of about 65% of sand fly vector population, and this needs to be tailored to who contributes most (Miller et al., 2014). Similar studies focusing on asymptomatic infection are needed urgently.

#### 3.2.5 Transmission and Infection

Current pitfalls in transmission and infection include factors relating to the sand fly vector, the parasite and the host. Characteristics and determinants of sand fly transmission have been described for each region; a full description is beyond the scope of this paper (Bern et al., 2010; Elnaiem, 2011; Miller et al., 2014; Aklilu et al., 2017).

The epidemiology of sand fly distribution and habitat and contribution to transmission needs regional precision as to spatial and seasonal fluctuations, and the firm identification of the reservoir.

Sand fly competence may be influenced by genetic exchange with leishmania parasites and co-infection; infection with other phleboviruses or other microbiota may be important and could be targeted for intervention. Sand fly saliva has among other, immunomodulatory properties and multiple bites may result in increasing degree of immunization in the naive host with implications for parasite load in the host and clinical implications including asymptomatic infection or clinical VL (Lestinova et al., 2017). Vaccination studies could explore the option of a combined vector salivary protein with a Leishmania antigen (Kamhawi et al., 2014).

Both in the ISC and in East Africa there is no consensus on the existence of an animal reservoir. While leishmania parasites have been demonstrated in animals, no evidence exist that these animals contribute to transmission (Bourdeau et al., 2020; Kushwaha et al., 2021).

Xenodiagnostic studies may be considered the gold standard for determining infectiousness. However, standardization of protocols is needed. Interpretation of findings should take into account that xenodiagnosis may not necessarily mimic the natural situation as sand fly competency in the wild may be different from what is found in the lab. Other variables that are difficult to control are parts of the body exposed to sand fly bites and the natural feeding timing of sand flies during the day (Singh et al., 2020). While studies in transmission including xenodiagnosis on PKDL and asymptomatic infection are well under way in the ISC, East Africa lags far behind; also here there is a need for regional precision.

Modelling is a powerful tool in precision medicine and global health, and key in decision making in control programs, such as the KAEP. There is no modelling effort for East Africa. Parameters of the model may relate to infection by sand flies (exposure, indoor or outdoor transmission, house-hold transmission, seasonality), outcome of infection (active VL, ex-VL, PKDL [macular vs polymorphic, interval after VL, self-cure or not], asymptomatic infection, VL-HIV co-infection), and infectivity of infected individuals to sand flies. This includes the presence of an animal reservoir. Issues on (life-long) acquired immunity, the effect on persisting parasites and transmissibility are important determinants. It follows that all parameters should be described in as much detail as possible as to their contribution. Also here regional precision is needed to be able to target interventions. These may focus on relatively few super-spreaders such as VL patients or HIV-VL co-infected patients during epidemics, while during inter-epidemic periods asymptomatic (high numbers, probably low infectivity) and/or PKDL patients (low numbers, high infectivity) may play a role in new outbreaks. Clearly these efforts should take into account the introduction of a vaccine, active or passive case detection, sand fly control by insecticide residual spraying, bed nets, or combinations, etc. (Chapman et al., 2018a; Chapman et al., 2018b; Le Rutte et al., 2019)

## 4 Summary

Precision medicine and global health offer the possibility to examine and to apply genomic and non-genomic tools in control of VL caused by *L. donovani*, with focus on the five priorities (diagnosis, treatment, PKDL, asymptomatic infection and transmission), all of which are interlinked by the immune responses. These new and powerful tools have contributed considerably to our understanding of the pathophysiology and epidemiology of VL and have the potential to change and revolutionize our approach in research and control. (**Table 1**).

**Table 1:** Priorities for precision in VL and PKDL research.

**Diagnosis**	Genomic tools
	○ Metagenomics – identification of co-infection
	○ Transcriptomics – genetic determinants (‘gene signature’) for diagnosis and outcome
	○ Nanodiagnostics – superior accuracy
	Adapted tools
	○ Use of multiplex PCR in differential diagnosis
	○ Use of panel of cytokine markers in diagnosis and as a biomarker.
	○ Re-development of the LST that is safe and antigenic in all endemic areas
	Priorities - general
	○ Development of PCR based diagnosis under field conditions
	○ Explore combination of tests, e.g. an anti- and a pro-inflammatory marker.
	○ Evaluation of diagnostic tests in longitudinal studies rather than in stored samples to assess PPV and NPV
	○ Explore paired strains of VL and PKDL patients vs. VL strains of patients who do not develop PKDL
	Priorities - biomarker
	○ Focus on immune parameters that reflect cell-mediated immunity, e.g. a cytokine ratio
	○ Relationship between parasite detection tests (PCR, antigen tests) and immune responses during and after treatment
	○ Serological tests are unlikely to be useful as a biomarker as a sole test
**Treatment and immune manipulation**	Treatment target
	○ Shift of focus from parasitological killing to assessment of drug- induced protective immune responses including those mediated by the drug
	○ Examine the benefit and risk of non-sterile cure vs sterile cure, at individual and population level
	Evaluation of treatment
	○ Evaluate treatment efficacy in relation to parasite load, co-infection and malnutrition, for individual precision
	○ Evaluate treatment efficacy and safety in all populations including (pregnant) women and children
	○ Develop drugs in parallel with biomarker (theragnostics)
	○ Explore population pharmacokinetic modelling for anti-leishmania drugs in each endemic area
	○ Describe genetic factors (host and parasite derived) that predict efficacy and/or toxicity of anti-leishmania drugs in endemic regions
	○ PK/PD studies in all drug studies including penetration in the skin as a parameter to prevent and /or treat PKDL
	○ Nanotechnology to detect drug resistance
	Immune manipulation
	○ Evaluation of immunomodulators
	○ Prophylactic vaccination for VL; prophylactic and therapeutic vaccination for PKDL
	Cost-effectiveness of interventions
	○ Use of pharmaco-economics to assess cost efficiency (e.g. drug treatment vs. vaccination)
**PKDL**	Priorities in diagnosis and biomarker
	○ Use of multiplex PCR in differential diagnosis
	○ Explore the use of artificial intelligence (deep learning) to recognize and (differential) diagnose at field level
	○ Explore immunological parameters, e.g. a ratio of a anti- and pro-inflammatory cytokine
	○ Explore 3 -dimensional scanning as a biomarker
	Priorities in treatment
	○ Short, ambulatory, safe and effective treatment with aim of pushing the immune response towards a cure profile
	○ Explore differences in PK/PD in treatment of PKDL vs treatment of VL, including drug levels in the skin
	○ Explore use of biologicals
	○ Explore use of immune modulator or prophylactic/ therapeutic vaccine
	Priorities in prevention
	○ Optimal drug treatment for VL with lowest possible PKDL rate
	○ Explore pathophysiological trigger for late occurrence of PKDL in the ISC - intercurrent infection (helminths), loss of immunological memory (as in measles), other factors
	○ Prophylactic vaccine to be used in combination with VL treatment
**Asymptomatic infection**	Definition
	○ Describe uniform definition
	○ Describe determinants (host, parasite, vector)
	○ Define robust markers for progression to VL that can be used in the field
	Epidemiology
	○ Description (incidence), characterization and infectivity in early and late phase of outbreaks and in endemic transmission
	○ Determine infectivity in all endemic areas
**Infectivity and transmission**	Infectivity
	○ Establish uniform protocols (PCR, sand fly bites)
	○ Describe infectivity in the whole spectrum of VL and PKDL, including HIV co-infection
	○ Describe changes in infectivity in the ISC after the start of treatment
	○ Determine infectivity in Africa in papular /nodular and macular PKDL, in early vs late development of PKDL, in acute vs chronic PKDL, according to age group
	Transmission
	○ Examine a possible animal reservoir in both East Africa and the ISC
	○ Further develop model for monitoring in ISC
	○ Develop model for interventions in Africa

Genomics offers new tools in diagnosis based on the interaction between parasite, host and vector, of which transcriptomics seems most promising. While these tools still need to be developed and evaluated further for applicability in an LMIC setting, they have the potential to replace traditional tools such as serological diagnosis with improved accuracy, while at the same time providing information on protective immune response. Similar advantages may be expected in treatment when the immune response is linked to the genomics in endemic populations, such as transcriptomic profiles. The increased understanding of protective immune responses may very well lead in the long term to priority of vaccine development in relation to drug development, while combinations such as immunochemotherapy may be of medium term benefit. In addition, non-genomic tools (e.g. the LST) or further development of existing (molecular) tools (e.g. multiplex platforms for PCR and cytokines) offer rapid assessment of the immune responses.

Description of genomic and non-genomic tools is relevant to PKDL and asymptomatic infection to describe risk factors with relation to host, parasite and vector. Here the understanding of immune responses is key, including potential triggers such as intercurrent infection, or factors relevant for protection (in VL to develop PKDL) or progression VL clinical disease (in asymptomatics). In transmission, the genetic exchange between vector and parasites or symbionts and effect of vector saliva on host immune responses when exposed is likely to influence vaccine development.

Key in both VL and PKDL is the understanding and manipulation of the (developing) immune response that is determined by, among other, genetic factors in the host, the parasite and the sand fly. (**Figure 1**) The immune response acts as a major final common pathway in the 5 priorities (**Table 1**). In this respect, a change in paradigm may be needed from classical microscopical diagnosis and parasite-killing drugs, to immune-based diagnosis and biomarkers, drugs in combination with immune modulation, and vaccination.

## 5 Conclusion

Research efforts in leishmaniasis are often fragmented, vertical and not well coordinated; for example, development of drugs, diagnostic tools, or vaccines are often done independently. Studies on basic science, pathophysiology and immune responses often do not run in parallel with clinical research. The lack of a holistic approach causes inefficiency, unnecessary delays and inefficient use of funds.

There is a need to continually explore the landscape, and include and use innovative approaches that arise in the whole field of internal medicine and paediatrics, radiology, laboratory and basic science, etc. Studies on cross-cutting issues with other tropical conditions could lead to better efficiency e.g. the study of asymptomatics and immune responses among the kinetoplastic diseases (human African trypanosomiasis and Chagas’ disease). Genomic tools have the potential to replace traditional tools in pathophysiology, management and control, offering precision in each endemic area. Precision in VL (in clinical medicine as well as public health) should currently be tailored to the (intra-) regional level given the heterogeneity of hosts, parasites and sand flies. Tailored treatment is already the case in HIV-VL co-infected patients, and should apply to management of VL and PKDL in the future. The next step would be to strengthen the on-going research efforts for precision for groups (e.g. children, women, pregnancy, VL-HIV co-infection). Once this has been achieved, further development for precision at the individual level will not be unrealistic.

## Author Contributions

The author confirms being the sole contributor of this work and has approved it for publication.

## Conflict of Interest

The author declares that the research was conducted in the absence of any commercial or financial relationships that could be construed as a potential conflict of interest.

## Publisher’s Note

All claims expressed in this article are solely those of the authors and do not necessarily represent those of their affiliated organizations, or those of the publisher, the editors and the reviewers. Any product that may be evaluated in this article, or claim that may be made by its manufacturer, is not guaranteed or endorsed by the publisher.
